# Genomic and phenotypic characterization of Antarctic *Pseudomonas/Aquipseudomonas* sp. strains with cold-active antifungal activity against *Botrytis cinerea*

**DOI:** 10.3389/fmicb.2026.1969102

**Published:** 2026-09-23

**Authors:** Nicolás Plaza, Matías Poblete-Morales, Katherine García, Marlen Barreto, Sebastián Lagos-Moraga, Evelyn Silva-Moreno, Ítalo M. Urrutia, Gino Corsini

**Affiliations:** 1 Instituto de Ciencias Biomédicas, Facultad de Ciencias de la Salud, Universidad Autónoma de Chile, Santiago, Chile; 2 Fundación Arturo López Pérez OECI Cancer Center, Centro de Investigación e Innovación en Cáncer, Santiago, Chile

**Keywords:** Antarctic bacteria, antifungal activity, biological control, *Botrytis cinerea*, postharvest, *Pseudomonas*, psychrotolerant

## Abstract

Gray mold, caused by the psychrophilic necrotroph *Botrytis cinerea*, is a leading cause of postharvest losses, and its control is increasingly compromised by the spread of fungicide-resistant field populations and by the failure of conventional biocontrol agents to operate at cold-chain temperatures. Antarctic soils are a rational yet underexploited source of cold-active antagonists. Here we isolated cultivable bacteria from the rhizosphere of *Deschampsia antarctica* (King George Island) and screened them against *B. cinerea* at 22 °C and 0 °C. Of 57 bacterial isolates, 44 antagonized the pathogen at 22 °C but only 28 retained activity at 0 °C. Five isolates with indistinguishable rep-PCR fingerprints of the genus *Pseudomonas* (*Pseudomonas fluorescens* group) inhibited mycelial growth by ≥70% at low temperature and were equally effective against fenhexamid-resistant and multidrug-resistant strains, whereas the mesophilic references *Bacillus subtilis* and *Bacillus amyloliquefaciens* lost all activity at 0 °C. Polyphasic and genome-based characterization of the representative strain AN3A02 placed it within the recently proposed genus *Aquipseudomonas* (GTDB-Tk) and identified it as a putative novel species (ANI 94.89% and dDDH 57.9% against the closest type strain, *P. Yamanorum*). Genome mining predicted 10 biosynthetic gene clusters consistent with a multifactorial antagonism combining lipopeptides, high-affinity siderophores and volatile compounds. In contact-free assays, AN3A02 volatiles suppressed mycelial growth and the formation of resistance and reproductive structures at 0 °C and, on inoculated blueberries and table grapes stored under simulated cold-chain conditions, reduced gray-mold incidence below the level achieved by AN SO₂ emitter. These cold-adapted Antarctic *Pseudomonas*/*Aquipseudomonas* strains are promising candidates for the biological control of fungicide-resistant gray mold along the fruit cold chain.

## 1 Introduction

The growing global demand for food has driven the expansion of large-scale monocultures managed through the intensive use of synthetic chemical fungicides, whose sustained misuse has selected fungicide-resistant populations across plant, animal, and human pathogens, an emerging threat to agricultural productivity, food security, and human health (Fisher et al., 2018). This demand coexists with a consumer trend towards healthier and more natural foods, which has narrowed tolerance for agrochemical residues in fresh produce (Nazzaro et al., 2017).

Among the most prevalent phytopathogens, *Botrytis cinerea* Pers. (teleomorph *Botryotinia fuckeliana*) is the causal agent of gray mold, a filamentous, ubiquitous, psychrophilic, necrotrophic, and polyphagous fungus of worldwide distribution (Benito et al., 2000; Fillinger and Elad, 2016; Ullah et al., 2024). Its infective unit is the conidiospore, produced on virtually any plant material and dispersed over long distances by air currents. Once it reaches the host, the infection cycle induces cell death and the eventual rotting of commercially important fruit, supported by a broad arsenal of virulence factors (Choquer et al., 2007). It affects at least 240 crop species, including grapevine, tomato, and berries (Jarvis, 1977), and its lack of host specificity makes many of them biological reservoirs that sustain its prevalence in production areas (Agrios, 2005; Fernández-Baldo et al., 2011).

Gray mold develops during crop growth and throughout storage and transport, which is critical for Chile, the leading fresh fruit exporter of the Southern Hemisphere, where over 60 species are shipped to more than 100 countries, and table grape and blueberry occupy 47,084 and 16,121 hectares (Gabler et al., 2003; Muñoz, 2018). The distance to the main markets imposes storage and transport periods of 20 to 90 days close to 0 °C, up to 100 days from harvest to consumer for the table grape cv. Red Globe (Judet-Correia et al., 2010). Berries are particularly vulnerable because of their high water and sugar content, and *B. cinerea* grows comfortably at the temperatures at which they are stored (Guentzel et al., 2010). Despite rapid cooling and controlled atmospheres of sulfur dioxide, losses of 15–40% in the field and up to 30% in postharvest are attributed to this pathogen in Chile, and apparently healthy fruit can rot suddenly en route to distant markets (Liu et al., 2007; Latorre et al., 2015; Rahman M. U. et al., 2024; Lopes et al., 2017).

The traditional strategy relies on preharvest synthetic fungicides from families such as dicarboximides, phenylpyrroles, anilinopyrimidines, hydroxyanilides, carboxamides, strobilurins, and triazoles. Their use over more than 50 years has eroded their effectiveness by selecting resistant isolates (Cruz, 2004; Jacometti et al., 2010; Nicot et al., 2016). Resistance in *B. cinerea* has been reported against 15 classes of systemic and protectant fungicides (Hahn, 2014), and multidrug-resistant field populations combining several mechanisms are established (Leroux et al., 2002; Kretschmer et al., 2009; Shao et al., 2021), botryticides with single-site modes of action being particularly prone to this erosion (Latorre et al., 1994). In parallel, agrochemical residues face increasingly strict limits, set individually and as cumulative totals, depending on the destination market, leaving growers without effective chemical alternatives (Fisher et al., 2018; Lopes et al., 2017).

Biological control is one of the most promising alternatives; antagonistic microorganisms are perceived as more environmentally benign than conventional fungicides, and their multifactorial modes of action are thought to reduce the risk of resistance development (Nicot et al., 2016). The most studied antagonists include fungi of the genera *Trichoderma*, *Ulocladium,* and *Gliocladium*, bacteria of the genera *Bacillus* and *Pseudomonas*, and yeasts of the genera *Pichia* and *Candida* (Vargas et al., 2012; Rahman F. U. et al., 2024; Nicot et al., 2016). In Chile *Trichoderma* has been used against *B. cinerea* in table grapes, but the control achieved in the field is only moderate (Latorre, 2007), and commercial products based on *B. subtilis*, which also promote plant growth and solubilize phosphates (Swain and Ray, 2009), do not match the efficacy of chemical mixtures (Senthil et al., 2011). The decisive limitation, however, is temperature; these formulations are designed for preharvest use and lose efficacy in the cold chain, whereas resistant field isolates of *B. cinerea* grow comfortably below 4 °C. Control of gray mold at 0 °C, which is also safe for consumers and the environment, therefore, remains an unmet need.

Antarctic soils offer a reliable source of antagonists capable of operating under these conditions. Approximately 99% of the microbial species of extreme environments remain uncultivated and are a promising reservoir of new antimicrobial compounds (Lewis, 2012), and the lower thermal limit for life is below −20 °C (Tindall, 2004; Mykytczuk et al., 2013). Long regarded as sterile, the coldest and driest continent hosts most of its biological diversity in inconspicuous microbial groups, particularly bacteria, whose soil richness is influenced by moisture and by the presence of plants and birds (Convey and Smith, 2006; Teixeira et al., 2013; Lambrechts et al., 2019). Antarctic soils remain comparatively unexplored with respect to aquatic environments (van Trappen et al., 2002; Velasco-Castrillón et al., 2014), yet their cultivable bacteria already support applied research, *Pseudomonas* isolates degrade diesel and acrylamide and reduce molybdenum (Shukor et al., 2009a; Shukor et al., 2009b; Ahmad et al., 2013; Núñez-Montero et al., 2020), actinobacteria inhibit *Candida albicans* and *Staphylococcus aureus* (Lee et al., 2012; Bruna et al., 2024), soil bacteria from Livingston Island antagonize phytopathogens such as *Erwinia amylovora* (Encheva-Malinova et al., 2014), and cold-active enzymes of value have been characterized (Ali et al., 2013; Guo et al., 2009). Reports of Antarctic bacteria producing antifungal metabolites are, however, scarce (D'Amico et al., 2006). Although the volatile fraction of an Antarctic *Pseudomonas* isolate has recently been chemically characterized and shown to inhibit *B. cinerea* on blueberries (Ulloa et al., 2023), the taxonomic identity of such isolates, the genomic basis of their antagonism, and the extent to which cold-active activity against fungicide-resistant strains is shared across them have remained largely unexplored.

In the present study, we isolated and characterized cultivable bacteria from rhizosphere soil of *Deschampsia antarctica* Desv., one of only two vascular plants native to the Antarctic continent, and evaluated their antifungal activity against *B. cinerea*, including fungicide-resistant strains, using dual culture assays at both 22 °C and 0–2 °C. We further characterized the most active isolates using a polyphasic approach that combined 16S rDNA phylogeny, repetitive sequence genotyping, phenotypic profiling, and whole-genome analysis of a representative strain.

## 2 Materials and methods

### 2.1 Soil sampling and bacterial isolation

Rhizosphere soil samples were collected from three sites in the periphery of the Polish Henryk Arctowski research station on King George Island (62°09′41″S, 58°28′10″W) during the 2018 Antarctic summer. Sampling was performed near stands of the vascular plant *D. antarctica* Desv., to a depth of no more than 10 cm (surface soil layer). For the recovery of the cultivable aerobic fraction, soil was plated directly onto solid nutrient agar (NA; 0.5% peptone, 0.3% yeast extract, 0.5% NaCl, 1.5% agar). In parallel, 1 g of soil was suspended in 9 mL of sterile saline (0.85% NaCl), serially diluted 10-fold, and plated on NA, potato-dextrose agar (PDA), water agar, and malt-yeast agar; plates were incubated at 18 °C for up to 2 weeks, and colonies were picked as they appeared. To recover spore-forming taxa, the protocol of Larkin and Stokes (1967) was additionally applied, in which soil suspensions were heated at 80 °C for 15 min, inoculated into tryptic soy broth, and incubated for 2 weeks at 0 °C prior to plating. Purified isolates were maintained as glycerol stocks (10% v/v) at −80 °C.

### 2.2 *Botrytis cinerea* strains

Three *B. cinerea* strains differing in their reported responses to fungicides were used (Table 1): the reference strain B05.10, with no identified resistance; strain 7 ac, resistant to fenhexamid; and strain PN2, resistant to fenhexamid, iprodione, pyrimethanil, and boscalid. The fungicide-resistance profiles of the native isolates 7 ac and PN2 were previously characterized by our group using discriminatory fungicide concentrations (Olea et al., 2019), whereas B05.10 is the internationally used reference isolate. Fungal strains were maintained on PDA and malt yeast agar and incubated in darkness at 22 °C for 5–6 days until confluence on 90-mm plates before each assay.

**Table 1:** *Botrytis cinerea* strains used in this study and their reported fungicide resistance.

Strain	Host	Reported resistance
B05.10	Grape	None identified (reference strain)
7 ac	Pear	Fenhexamid
PN2	Cherry	Fenhexamid, iprodione, pyrimethanil, boscalid

### 2.3 *In vitro* dual-culture antagonism assays

Antagonism was assessed using a dual-culture bioassay (Fokkema, 1978), following Guevara-Avendaño et al. (2018), with modifications. A 5-mm agar plug bearing actively growing *B. cinerea* mycelium was placed at the center of an NA plate, and 10 μL of a fresh bacterial suspension (optical density (OD) at 620 nm = 0.1) was inoculated at each of the four cardinal points, 20 mm from the center. One cardinal point and a separate plate were left uninoculated as growth controls. *Bacillus subtilis* and *Bacillus amyloliquefaciens* were included as reference antagonists. Plates were incubated at 22 ± 1 °C for 3 days and at 0 ± 2 °C for up to 14–21 days, and the mycelial radius was measured daily. The percentage of inhibition was calculated as [(*R* − *r*)/*R*] × 100, where *R* is the mycelial radius of the control and r the radius of the mycelium facing the bacterial isolate (Skidmore and Dickinson, 1976). Each assay was performed with three independent biological replicates, each including three technical replicates.

### 2.4 16S rDNA amplification and phylogenetic analysis

Genomic DNA of the most active isolates was extracted with InstaGene Matrix (Bio-Rad) according to the manufacturer’s instructions. The 16S rDNA gene was amplified by PCR with primers fD1-rD1 (Weisburg et al., 1991), purified using Wizard SV Gel and PCR Clean-Up (Promega), and sequenced from both ends (Macrogen Inc., Korea). Sequences were assembled and curated in SeqMan and MEGA X (Kumar et al., 2018) and compared against the EzBioCloud database (Yoon et al., 2017). Partial 16S rDNA sequences were aligned with those of related species, and a phylogenetic tree was reconstructed by neighbor-joining with 1,000 bootstrap replicates in MEGA X. To refine the placement within the genus, a multilocus sequence analysis (MLSA) including the 16S rRNA, *gyr*B, *rpo*B, and *rpo*D genes was performed as recommended for *Pseudomonas* (Mulet et al., 2010; Gomila et al., 2015).

### 2.5 Repetitive-sequence genotyping (rep-PCR)

REP, ERIC, and BOX-PCR (collectively rep-PCR) were used to genotype the isolates identified as inhibitors of *B. cinerea*. Total DNA was adjusted to 100 ng μL^−1^ and amplified with the primer sets and cycling conditions recommended for *Pseudomonas* (Versalovic et al., 1991; Louws et al., 1994) using GoTaq Green Master Mix (Promega). Amplification products were resolved by horizontal electrophoresis (100 V, 40 min) in 2% agarose in 1 × TAE stained with GelRed (Biotium), alongside a GeneRuler 100 bp Plus DNA Ladder (Thermo Scientific), and visualized under UV (Gel Doc XR, Bio-Rad).

### 2.6 Phenotypic characterization and electron microscopy

Isolates were characterized by classical microbiological techniques, including colony morphology, Gram staining, motility, catalase and oxidase reactions, nitrate reduction, ornithine decarboxylase and urease activity, citrate utilization, H₂S production, and growth on LB supplemented with 0–5% NaCl (Duveiller et al., 1997). Enzymatic profiles of representative isolates were determined using the API-ZYM galleries (bioMérieux) according to the manufacturer’s instructions. Hemolytic activity was scored on Müller-Hinton agar supplemented with 5% sheep blood (48 h, 28 °C). Siderophore production was assessed by the chrome-azurol-S (CAS) assay (Schwyn and Neilands, 1987) under standard conditions (28 °C), phosphate solubilization was evaluated on Sperber medium, and indole-3-acetic acid was detected with the Salkowski reagent (Gordon and Weber, 1951). Cell size and flagellation of the representative strain AN3A02 were examined by transmission electron microscopy (HT7700 TEM, Hitachi) operated at 80 kV.

### 2.7 Taxonomic assignment and global phylogenomic analysis

The genome sequence of strain AN3A02, previously reported by Poblete-Morales et al. (2020), was evaluated to establish its detailed taxonomic standing and phylogenomic relationships. Standardized taxonomical assignment was performed using the Genome Taxonomy Database Toolkit (GTDB-tk) v2.7.2 (Chaumeil et al., 2022), composed of 120 single-copy bacterial marker genes (bac120). The concatenated amino acid sequence alignment was filtered using default GTDB masks and used to perform a Maximum-likelihood (ML) phylogenomic tree with FastTreeMP v2.2.0 (Price et al., 2010), under the WAG amino acid substitution model with Gamma-distributed rate heterogeneity among sites. The resulting tree topology was utilized to establish the placement of strain AN3A02 within the *Pseudomonadaceae* family and to select the 24 most closely related reference genomes for downstream species boundary demarcation.

### 2.8 Average nucleotide identity (ANI) and *in silico* digital DNA–DNA hybridization (dDDH) analysis

Pairwise ANI calculations were conducted to evaluate species boundaries between strain AN3A02 and the 24 most closely related reference genomes. Analysis was performed using the pyani-plus (v1.0.1) suite via the MUMmer algorithm (Pritchard et al., 2016). Pairwise ANI percentage hierarchical clustering was performed using the pheatmap package v1.0.13 (Kolde, 2025) based on Euclidean distances and Unweighted Pair Group Method with Arithmetic Mean (UPGMA/average linkage) in Rstudio (v2026.7.1.147) using R (v4.6.1). Strains sharing an ANI value below the standard 95–96% species-boundary threshold were considered to represent distinct species (Richter and Rosselló-Móra, 2009).

*In silico* dDDH was performed with the Genome-to-Genome Distance Calculator 3.0^1^ against the type strain *Pseudomonas yamanorum* LMG 27247, and results were computed using formula d4 (Meier-Kolthoff et al., 2013). A dDDH value below the standard 70% threshold was considered indicative of species-level distinction (Meier-Kolthoff et al., 2013).

### 2.9 *In silico* secondary-metabolite mining

Biosynthetic gene clusters (BGCs) potentially encoding secondary metabolites were predicted from the *Pseudomonas* sp. AN3A02 genome using antiSMASH v8.0.4 (Blin et al., 2025) under ‘strict’ detection parameters. The KnownClusterBlast feature was enabled to evaluate sequence homology and similarity against characterized reference pathways in the Minimum Information about a Biosynthetic Gene Cluster (MIBiG) database (Zdouc et al., 2025).

### 2.10 Volatile-mediated antagonism assay

The activity of volatile compounds was assessed in sealed two-compartment Petri plates (I-plates). One compartment contained potato dextrose agar inoculated with a 5-mm mycelial plug of the *B. cinerea* strain to be tested, and the other contained nutrient agar (or LB) inoculated with 100 μL of an overnight bacterial suspension adjusted to an OD at 600 nm = 0.2. Plates were sealed with two layers of Parafilm M and incubated at 0 and 22 °C until the colony reached the plate edge. *B. cinerea* grown without the bacterium served as a control (Strobel et al., 2001). Colony diameter was recorded and expressed as a percentage of inhibition. In a long-duration variant simulating cold-chain storage, plates were incubated for 40 days at 0 °C, followed by 2 days at 22 °C, and the formation of resistance structures (sclerotia, conidiophores, and conidia) was examined by light microscopy after lactophenol blue staining.

### 2.11 Postharvest protection assay on fruit

The protective effect of the volatiles was evaluated on export blueberries and table grapes in collaboration with the INIA La Platina Postharvest Unit. Fruit was placed in hermetic plastic containers on a waxed grid that prevented contact between fruits, at >90% relative humidity and 0 °C. Three groups were established: (i) fruit inoculated near the pedicel wound with 2 μL of a 1 × 10^6^ conidia mL^−1^ suspension of the multidrug-resistant strain *B. cinerea* PN2 (20 fruits per container) and left untreated, (ii) the same inoculated fruit co-incubated with a 90 mm LB agar plate seeded with *Pseudomonas* sp. AN3A02 as a volatile-generating device, and (iii) inoculated fruit co-incubated with an SO₂-emitting sachet, the standard postharvest control. Blueberries were stored for 45 days and table grapes for 60 days at 0 °C, followed by 2 days at 20 °C, after which disease incidence, disease severity, and quality attributes were recorded. Disease incidence was defined as the number of fruits showing visible gray mold symptoms (mycelial development at the inoculation site) relative to the total number of fruits assessed per container, expressed as a percentage. Disease severity was scored for each fruit as the proportion of its surface colonized by *B. cinerea* and assigned to one of four categories (25, 50, 75, or 100% of the surface colonized); severity results are expressed as the percentage of fruit in each category per container. Fruit softening was scored on a 0–4 hedonic scale (0 = firm fruit). Each treatment comprised three replicate containers of 20 fruit each (*n* = 3), and a second evaluation was performed 2 days after opening the containers.

### 2.12 Statistical analysis

All quantitative assays were performed in three independent biological replicates, each comprising three technical replicates. Technical replicates were averaged within each biological replicate prior to analysis, and results are expressed as the mean ± standard deviation (SD) of the biological replicates. Mycelial-growth inhibition in the dual-culture assays against the three *B. cinerea* strains (B05.10, 7 ac, and PN2), including the mesophilic reference antagonists *B. subtilis* and *B. amyloliquefaciens*, is reported descriptively at both incubation temperatures (22 °C and 0 °C). For the postharvest assays, gray-mold incidence (proportion of decayed fruit) was compared among treatments (untreated control, AN3A02 volatiles, and SO₂ emitter) with Fisher’s exact test. Colonization-severity categories are reported descriptively as the percentage of fruit in each category. Differences were considered statistically significant at *p* < 0.05. All statistical analyses were performed using GraphPad Prism version 8.0.

## 3 Results

### 3.1 Isolation and *in vitro* screening of antifungal activity

A total of 76 cultivable microbial isolates were recovered from the rhizosphere soil of *D. antarctica* collected at three sampling sites. Of these, 57 Gram-typed, 16S rDNA confirmed bacterial isolates were screened for inhibition of mycelial growth of the *B. cinerea* reference strain B05.10 at 22 °C and 0 °C. Forty-four isolates showed some degree of antagonism, whether by competition for space or by the production of growth-inhibitory substances, whereas the remaining 13 isolates showed no detectable inhibition in the primary screen and were not quantified further, and 28 of these (63.6%) inhibited the pathogen at both temperatures. The percentage inhibition of the 44 antagonistic isolates and the two *Bacillus* reference strains is shown in Figure 1 and listed in Supplementary Table S1. Five isolates, AN2B06, AN2B07, AN3A02, AN3A05, and AN3B23, originating from two different sampling sites, inhibited mycelial growth by ≥70% at low temperature (0 ± 2 °C) and were selected for further study (Figure 1B).

**Figure 1:**
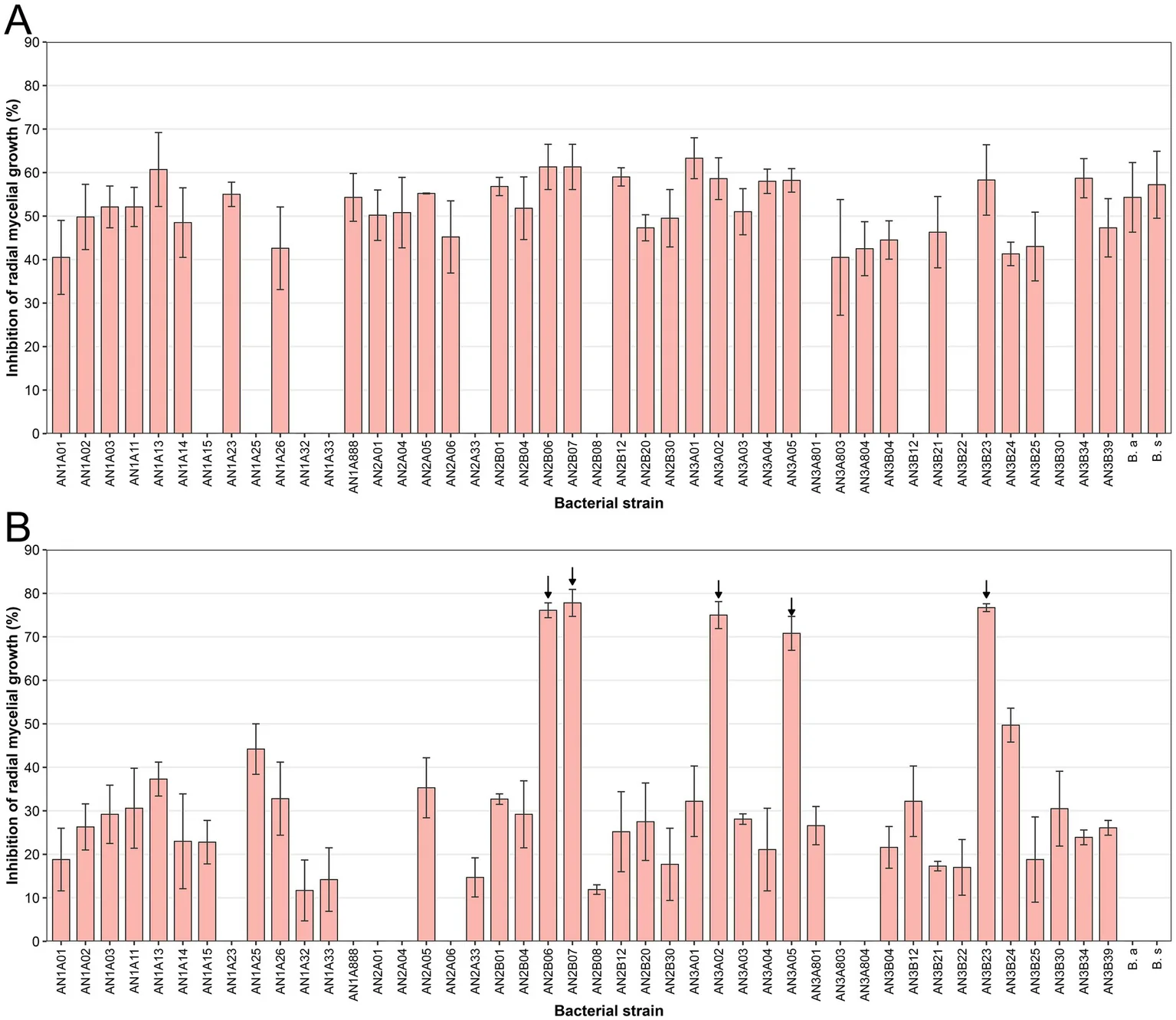
Percentage of inhibition of *B. cinerea* B05.10 mycelial growth in dual culture against Antarctic soil bacteria under two temperature regimes. **(A)** Co-culture at 22 ± 1 °C for 3 days. **(B)** Co-culture at 0 ± 2 °C for 14 days, arrows indicate the isolates with the highest inhibitory capacity selected for subsequent analyses. *B. subtilis* (B.s) and *B. amyloliquefaciens* (B.a) were included as reference antagonists. Values are means of three independent assays performed in technical triplicate; error bars represent the standard deviation. Complete numerical values (mean ± SD) for all isolates and reference strains are provided in Supplementary Table S1.

### 3.2 Antagonism against fungicide-resistant *B. cinerea* strains

The five selected isolates were tested against two native *B. cinerea* strains with documented resistance to synthetic fungicides, strain 7 ac (fenhexamid resistant) and the multidrug-resistant strain PN2, in addition to the reference strain B05.10. At both 22 ± 1 °C (Figure 2A) and 0 ± 2 °C (Figure 2B), the isolates inhibited the resistant strains to an extent comparable to that observed against the susceptible reference strain, indicating that their antagonism is independent of the target sites affected by the fungicides to which 7 ac and PN2 are resistant. In contrast, the mesophilic reference antagonists *B. subtilis* and *B. amyloliquefaciens*, which inhibited the three fungal strains by approximately 55% at 22 °C (Figure 2A), showed no detectable inhibition at 0 ± 2 °C (Figure 2A).

**Figure 2:**
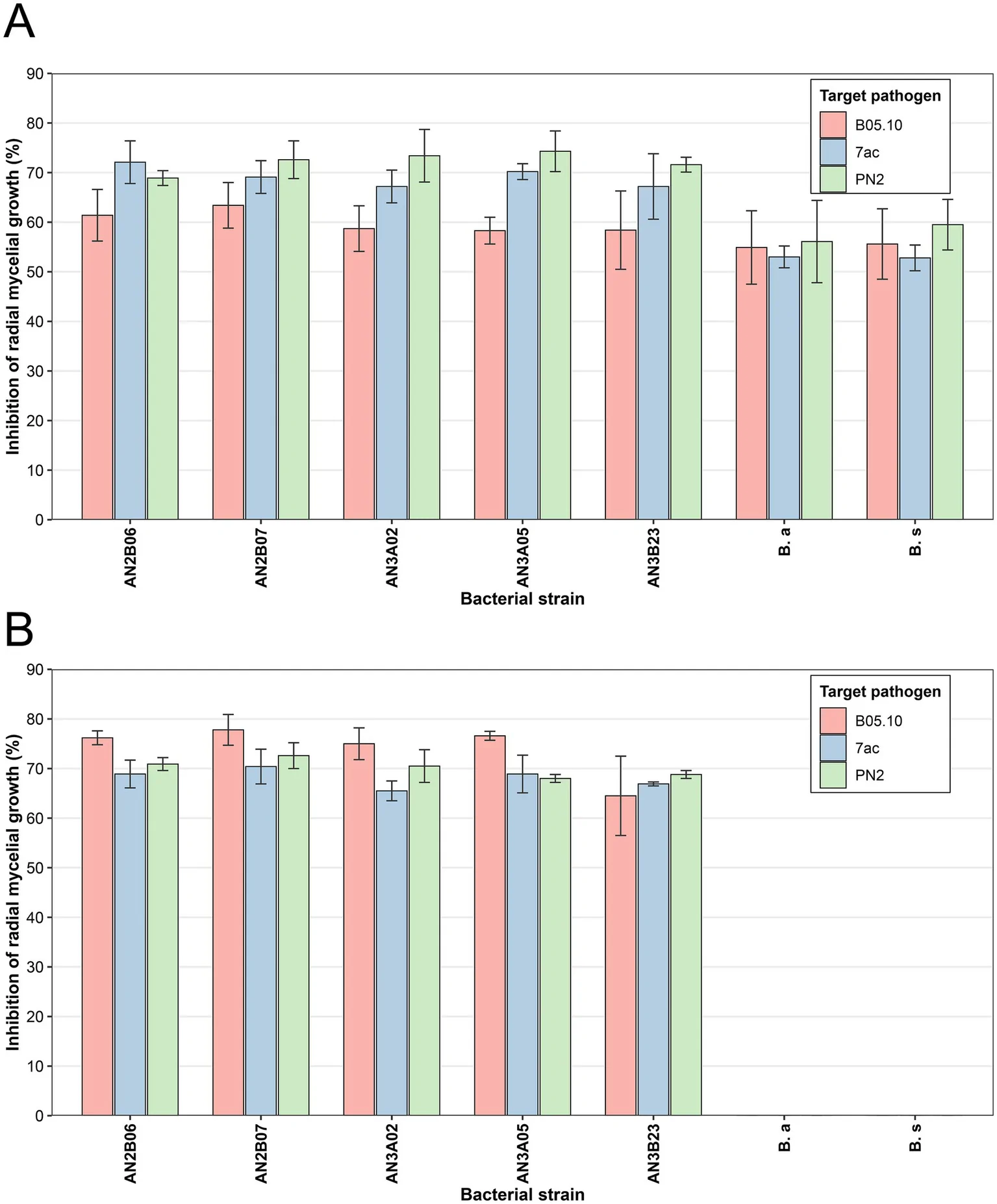
Percentage of inhibition exerted by Antarctic soil isolates against fungicide-resistant *B. cinerea* strains. **(A)** Incubation at 22 ± 1 °C for 3 days and **(B)** at 0 ± 2 °C for 14 days. B05.10, reference strain; 7 ac, fenhexamid-resistant strain; PN2, multidrug-resistant strain (fenhexamid, iprodione, pyrimethanil, and boscalid). *B. subtilis* (B.s) and *B. amyloliquefaciens* (B.a) were included as reference antagonists. Values are means of three independent assays in technical triplicate; error bars represent the standard deviation.

### 3.3 Molecular identification by 16S rDNA and phylogeny

To identify the five isolates at the genus level, the 16S rDNA was amplified and sequenced, yielding concatenated sequences averaging 1,433 ± 28 bp. All sequences shared ≥98.58% identity with *Pseudomonas yamanorum* 8H1ᵀ, a strain originally isolated from a subantarctic environment and belonging to the fluorescent lineage of the genus (Figure 3A). Given the high similarity, a phylogenetic reconstruction was performed with 16S rRNA sequences from members of the *Pseudomonas fluorescens* group. In the resulting tree, the five isolates clustered together in a single group with the *P. yamanorum* strain 8H1ᵀ and other members of the *P. gessardii* subgroup.

**Figure 3:**
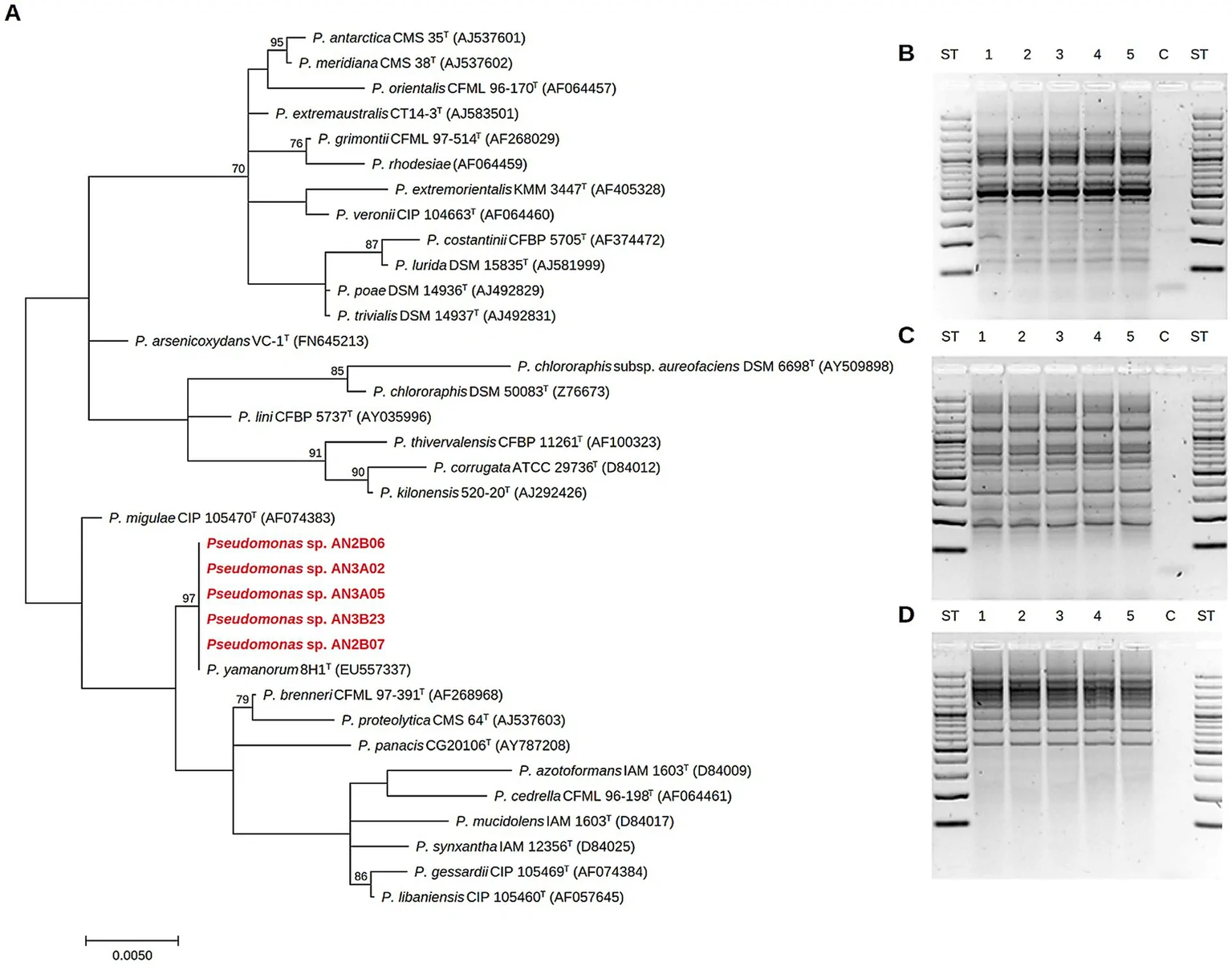
Molecular identification and clonal genotyping of the five Antarctic isolates with antifungal activity against *B. cinerea*. **(A)** Phylogenetic reconstruction based on 16S rDNA, maximum-likelihood topology (Kimura 2-parameter model) inferred for *Pseudomonas* species of the fluorescens group, with bootstrap values (>70%) based on 1,000 replicates, is shown at the nodes (scale bar: 5 substitutions per 1,000 nucleotides). **(B–D)** Repetitive-sequence genotyping (rep-PCR) of the same isolates. **(B)** ERIC-PCR, **(C)** REP-PCR, and **(D)** BOX-PCR. ST, GeneRuler 100 pb Plus. Line 1–5 isolates AN2B06, AN2B07, AN3A02, AN3A05, and AN3B23. C, no-DNA control. The five isolates show identical amplification profiles with the three primer sets.

### 3.4 Repetitive-sequence genotyping reveals indistinguishable genotypes among the five isolates

Repetitive-sequence PCR was used to examine the genetic relationships among the five isolates. Complex, reproducible fingerprints were readily obtained from the Antarctic DNA with the ERIC primers, and the three assays, REP, ERIC, and BOX-PCR, produced identical banding patterns across all five isolates (Figures 3B–D). The profiles were so similar that dendrogram construction was unnecessary; despite originating from different sampling sites, the isolates displayed indistinguishable rep-PCR fingerprints, consistent with a clonal or near-clonal population, because rep-PCR samples only part of the genome, this does not by itself establish complete genomic identity. This finding accounts for the decision to focus subsequent whole-genome characterization on a single representative strain rather than on all five isolates.

### 3.5 Phenotypic characterization and cell morphology

The five isolates with the highest anti-*Botrytis* activity were short, straight, Gram-negative, non-spore-forming, and motile rods. They grew over a temperature range of 0–30 °C, tolerated up to 4% NaCl, and fluoresced under ultraviolet light on King medium, consistent with membership in the *P. fluorescens* group (Table 2). Two representative isolates from different sampling sites, AN2B06 and AN3A02, showed identical API-ZYM profiles, both being positive for alkaline and acid phosphatase, esterase (C4), lipase (C8), leucine arylamidase, naphthol-AS-BI-phosphohydrolase, and β-glucosidase. All five isolates produced β-hemolysis on blood agar and tested positive on CAS plates, indicating siderophore production under standard assay conditions (28 °C). Transmission electron microscopy showed that the representative strain possessed a single polar flagellum and measured 0.9–1.2 μm in width by 1.9–2.8 μm in length (Supplementary Figure S1). The differential tests summarized in Table 2 clearly distinguished the representative isolate AN3A02 from its closest validly described relatives.

**Table 2:** Phenotypic and enzymatic characterization of the five Antarctic isolates with the highest inhibitory capacity against *B. cinerea*, compared with the closest reference species.

Characteristic	1	2	3	4	5	6	7*
Short rods	+	+	+	+	+	+	+
Gram reaction	−	−	−	−	−	−	−
Motility	+	+	+	+	+	+	+
Catalase	+	+	+	+	+	+	+
Oxidase	+	+	+	+	+	+	+
Spore formation	−	−	−	−	−	−	−
Growth range (°c)	0–30	0–30	0–30	0–30	0–30	18–42	4–35
NaCl tolerance (% w/v)	0–4	0–4	0–4	0–4	0–4	ND	0–6
Fluorescence (king medium)	+	+	+	+	+	+	+
Haemolysis (mh-blood)	*β*	*β*	*β*	*β*	*β*	*β*	ND
Siderophores (cas)	+	+	+	+	+	+	ND
Api-zym							
Alkaline phosphatase	+	ND	+	ND	ND	ND	+
Esterase (c4)	+	ND	+	ND	ND	ND	−
Lipase (c8)	+	ND	+	ND	ND	ND	−
Leucine arylamidase	+	ND	+	ND	ND	ND	+
Valine arylamidase	−	ND	−	ND	ND	ND	+
Acid phosphatase	+	ND	+	ND	ND	ND	+
Naphthol-as-bi-phosphohydrolase	+	ND	+	ND	ND	ND	+
*β*-glucosidase	+	ND	+	ND	ND	ND	−

Taxa: 1, Pseudomonas sp. AN2B06; 2, Pseudomonas sp. AN2B07; 3, Pseudomonas sp. AN3A02; 4, Pseudomonas sp. AN3A05; 5, Pseudomonas sp. AN3B23 (this study); 6, P. aeruginosa UA-01; 7*, P. yamanorum 8H1ᵀ (Arnau et al., 2015). +, positive; −, negative; ND, not determined.

### 3.6 Phylogenomic and species boundary delimitation of AN3A02

The genome of strain AN3A02 was previously reported by Poblete-Morales et al. (2020); however, its detailed taxonomic standing and phylogenetic relationships with related species remained unexplored. To establish its taxonomic classification, a standardized assessment was conducted using GTDB-Tk v2.7.2, which successfully identified 118 of the 120 bacterial marker genes, yielding a concatenated alignment of 5,010 amino acids. Global phylogenomic reconstruction alongside 1,264 reference genomes and 10 *Moraxellaceae* genomes selected as an outgroup, placed strain AN3A02 within the family *Pseudomonadaceae,* classifying it within the recently proposed genus *Aquipseudomonas* (Rudra and Gupta, 2024) (Figure 4A). This analysis established that strain AN3A02 lacks a species-level assignment among reference type strains and clusters in a tight monophyletic clade with *Pseudomonas* sp. ATCC PTA-122608 (Figure 4B) (García et al., 2021).

**Figure 4:**
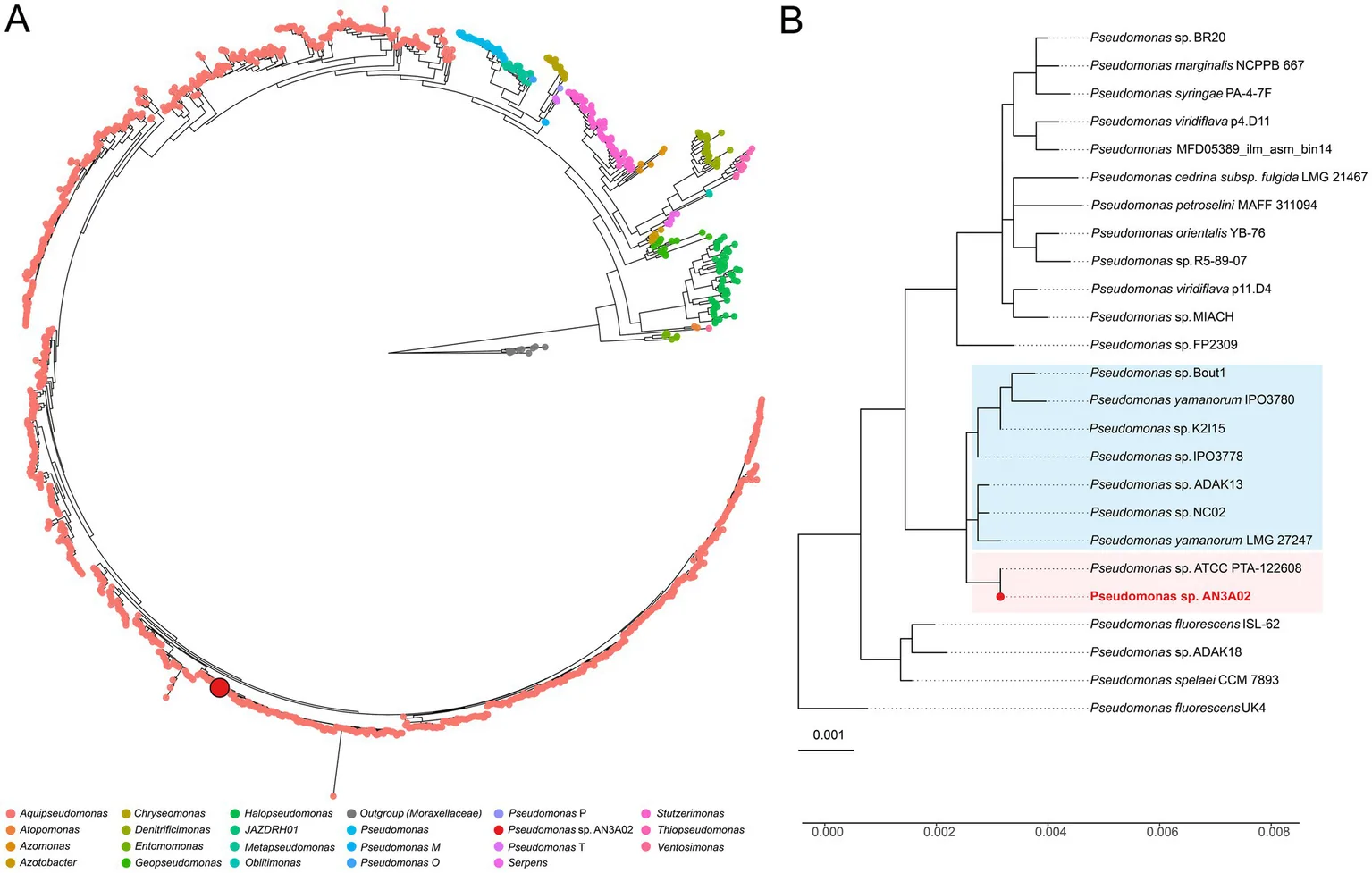
Global phylogenomic placement and detailed sub-clustering of strain AN3A02. ML tree reconstructed by FasTreeMP from a concatenated alignment of 118 single-copy bacterial marker genes (5,010 amino acids) identified via GTDB-Tk. **(A)** Global tree comprising 1,275 genomes, placing strain AN3A02 within the recently proposed genus *Aquipseudomonas*. **(B)** Subtree highlighting the 24 most closely related genomes to AN3A02, showing the tight monophyletic clade formed with *Pseudomonas* sp. ATCC PTA-122608 (red rectangle) and closest to *Pseudomonas yamanorum* cluster (sky blue rectangle).

To delineate species boundaries, an ANI matrix was computed against the 24 closest reference genomes selected from Figure 4B. The pairwise comparison revealed a 98.92% ANI between strain AN3A02 and *Pseudomonas* sp. ATCC PTA-122608 (Figure 5), comfortably exceeding the 95–96% species boundary threshold. Conversely, the closest validly named reference species was *Pseudomonas yamanorum* LMG 27247, exhibiting an ANI value of 94.89%, placing it at the borderline of species demarcation (> 95–96%).

**Figure 5:**
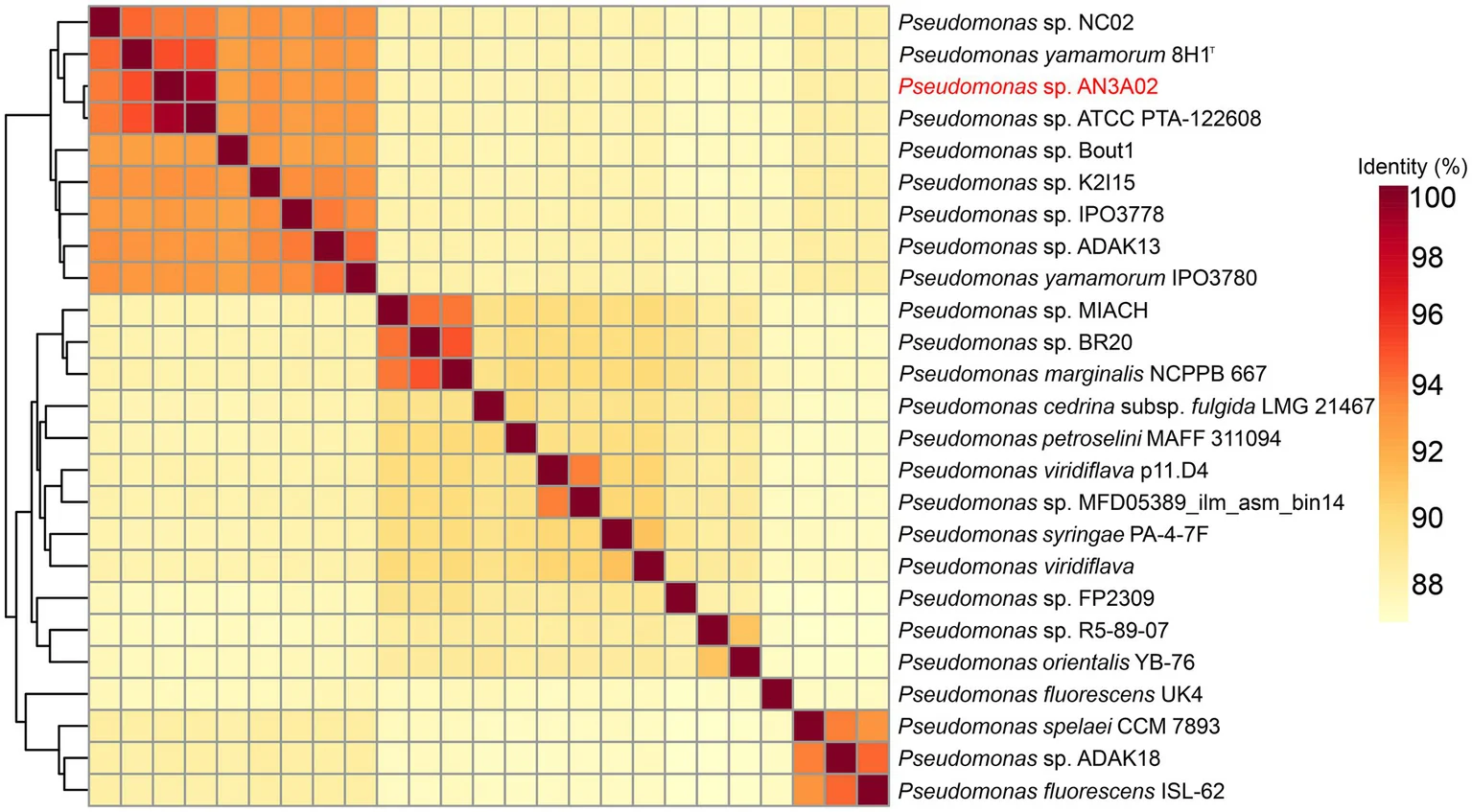
ANI heatmap comparing strain AN3A02 against 24 closely related reference genomes. Pairwise ANI values (%) displayed in each cell were computed using *pyani-plus*. The color scale indicates the percentage of global genomic identity. The left dendrogram illustrates hierarchical clustering based on Euclidean distance and average linkage (UPGMA).

To clarify whether strain AN3A02 and ATCC PTA-122608 belong to the *P. yamanorum* cluster, dDDH was performed via Genome-to-Genome Distance Calculator 3.0 using formula *d4*. Pairwise comparison against the type strain *P. yamanorum* LMG 27247 yielded a dDDH value of 57.9 and 57.8% for AN3A02 and ATCC PTA-122608, respectively, well below the standard 70% threshold required for species co-specificity.

Together, these data indicate that strain AN3A02 is closely related to the *P. yamanorum* cluster and likely constitutes a novel species of *Aquipseudomonas* with uncharacterized antifungal potential.

### 3.7 Secondary-metabolite biosynthetic potential

Genome mining of *Pseudomonas* sp. AN3A02, using antiSMASH v.8.0.2 and the MIBiG database, revealed a versatile biosynthetic landscape comprising 10 distinct BGCs distributed across its draft genome (Table 3). This biosynthetic machinery is consistent with the multifactorial antagonistic traits observed *in vitro*, in which direct antifungal activity against *B. cinerea* may be mediated, at least in part, by predicted Non-Ribosomal Peptide Synthetase (NRPS) clusters. Notably, Regions 16.1 and 17.1 encode complex NRPS clusters; the former displays medium sequence similarity to the tolaasin I/F biosynthetic cluster (Scherlach et al., 2013), whereas the latter represents an uncharacterized pathway potentially synthesizing novel peptide secondary metabolite (Table 3). In addition, two distinct iron-scavenging BGCs were detected: Region 1.3 encodes an NRP-metallophore cluster with medium similarity to pyoverdine SXM-1 (Guo et al., 2021), while Region 12.1 represents an additional NRPS cluster related to Pf-5 pyoverdine (Hartney et al., 2013) (Table 3). Regarding cold-temperature adaptation, Region 1.1 was predicted as an uncharacterized NAGGN-like (*N*-acetylglutaminylglutamine amide) BGC, a class of osmoprotectants typically involved in osmotic and cold-stress tolerance. Meanwhile, Region 1.2 encodes an arylpolyene cluster with low sequence similarity to known reference pathways, putatively contributing to lipophilic membrane protection against oxidative stress (Table 3). Together, these predicted BGCs suggested that *Pseudomonas* sp. AN3A02 has the genetic potential to produce a multifactorial combination of lipopeptides, high-affinity siderophores, volatile compounds, and stress-protective metabolites relevant to antagonism during cold storage. Of these predictions, siderophore production and volatile-mediated inhibition were experimentally corroborated in the present study (CAS assay and two-compartment assay, respectively), whereas the products and activities of the remaining clusters, such as the tolaasin-like NRPS, remain to be validated.

**Table 3:** *In silico* prediction of secondary-metabolite biosynthetic potential in the genome of *Pseudomonas* sp. AN3A02.

Region	BGC type	Position (bp)	Most similar known cluster	Similarity	Putative biological function
1.1	NAGGN	123,323–138,201	—	—	Osmoprotection/Cold-stress adaptation
1.2	Arylpolyene	1,174,473–1,218,048	Aryl polyenes	Low	Antioxidant/membrane stabilization
1.3	NRP-metallophore, NRPS	1,405,766–1,489,565	Pyoverdine SXM-1	Medium	Siderophore (Iron acquisition/CAS+)
1.4	Betalactone	1,569,240–1,593,107	—	—	Bioactive lipids/enzyme inhibition
4.1	Hydrogen-cyanide	123,447–136,177	Hydrogen cyanide	High	Volatile antifungal metabolite
12.1	NRPS	134,056–244,854	Pf-5 pyoverdine	Low	Secondary pyoverdine-like siderophore
14.1	Redox-cofactor	502,176–524,332	—	—	Cellular redox homeostasis
16.1	NRPS	1–26,664	Tolaasin I/Tolaasin F	Medium	Cyclic lipopeptide (Antifungal/Membranolytic)
17.1	NRPS	225,867–263,873	—	—	Uncharacterized non-ribosomal peptide
20.1	Hydrogen-cyanide	50,827–63,647	Hydrogen cyanide	High	Paralogous HCN biosynthetic operon

### 3.8 Volatile-mediated antagonism against *B. cinerea in vitro* and on fruit

To test whether the biosynthetic potential predicted in silico translates into antagonism, the volatile fraction of *Pseudomonas* sp. AN3A02 was assayed without physical contact with the pathogen. In sealed two-compartment plates, the bacterial volatiles markedly reduced the mycelial growth of *B. cinerea* and, in the long-duration cold assay (40 days at 0 °C), suppressed the formation of the resistance and reproductive structures (sclerotia, conidiophores, and conidia) that were abundant in the bacterium-free control (Figures 6A,B). The effect was then evaluated on inoculated fruit under simulated cold-chain conditions. On blueberries, the incidence of rot caused by the multidrug-resistant strain PN2 fell from 51.7% in the untreated control (31/60 fruit) to 1.7% (1/60 fruit) in fruit co-incubated with AN3A02 and was 66.7% (40/60 fruit) with the SO₂ emitter (three replicate containers of 20 fruit per treatment; *n* = 3), after 45 days at 0 °C plus 2 days at 20 °C. The reduction achieved by AN3A02 was significant relative to both the untreated control and the SO₂ emitter (Fisher’s exact test, *p* < 0.001), whereas the SO₂ emitter did not differ significantly from the control (*p* = 0.14). When infection did occur under the bacterial treatment, it remained restricted to the inoculation point (Figures 6C–E). For table grapes stored for 60 days at 0 °C plus 2 days at 20 °C, the incidence dropped from 100% (60/60 fruit) in both the untreated control and the SO₂-treated group to 30.0% (18/60 fruit) with AN3A02 (Fisher’s exact test, *p* < 0.001), with a marked reduction in colonization severity relative to the untreated and SO₂-treated groups (Figures 6F–H). Together, these assays indicate that the antifungal activity of AN3A02 is, at least in part, mediated by volatile compounds that remain active at 0 °C and are effective against fungicide-resistant *B. cinerea* on the surface of export fruit.

**Figure 6:**
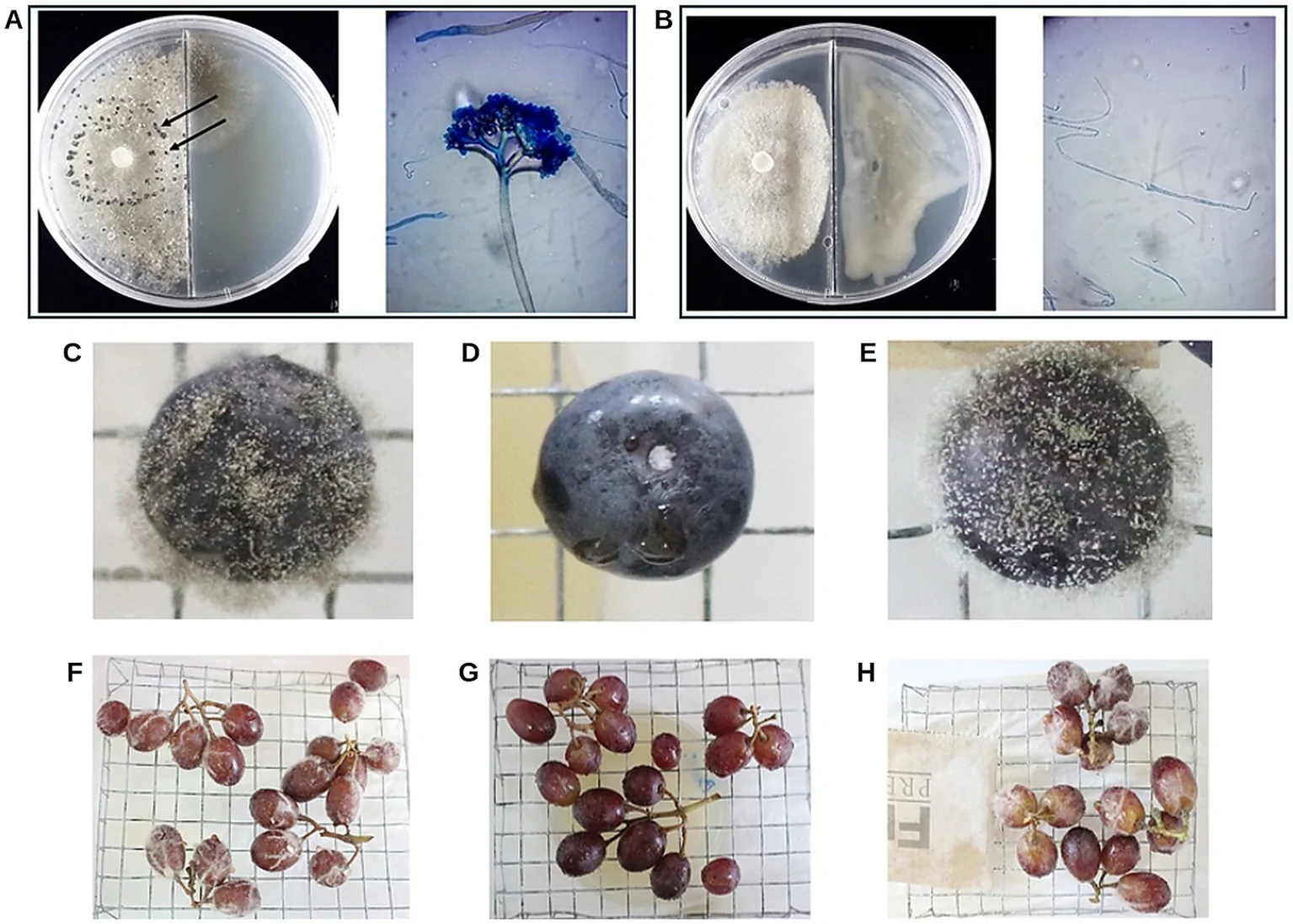
Volatile-mediated antagonism of *Pseudomonas* sp. AN3A02 against *B. cinerea in vitro* and on postharvest fruit. **(A,B)** *In vitro* two-compartment assay against *B. cinerea* B05.10, each shown as a macroscopic plate together with the corresponding light micrograph. **(A)** The fungus grown alone develops abundant mycelium with sclerotia (indicated by arrows) and conidiophores bearing conidia, whereas **(B)** co-incubation with AN3A02 markedly reduces growth and suppresses these resistance and reproductive structures. **(C–E)** Blueberries and **(F–H)** table grapes inoculated with the multidrug-resistant strain *B. cinerea* PN2 and stored under simulated cold-chain conditions (45 days at 0 °C plus 2 days at 20 °C for blueberries, 60 days at 0 °C plus 2 days at 20 °C for table grapes). **(C,F)** Untreated control, **(D,G)** fruit co-incubated with AN3A02 volatiles, and **(E,H)** fruit treated with an SO_2_ emitter. Each treatment comprised three independent replicate containers of 20 fruit each (*n* = 3). Disease incidence (proportion of decayed fruit) was compared among treatments using Fisher’s exact test (*p* < 0.05); colonization-severity categories are shown descriptively as the percentage of fruit in each category.

## 4 Discussion

For a long time after the discovery of the Antarctic continent in 1820, life was believed to be essentially absent from what was regarded as a sterile environment (Convey and Smith, 2006; Lambrechts et al., 2019). It is now understood that most Antarctic diversity resides in inconspicuous microbial groups, and cultivation campaigns over the last three decades have recovered members of only six bacterial phyla, among them *Proteobacteria* and *Firmicutes*. Although our study did not aim to resolve the phylogenetic affiliation of all 76 recovered isolates, morphological and partial 16S rRNA data confirmed the presence of at least two phyla in the rhizosphere of *D. antarctica* Desv., in agreement with previous surveys of Antarctic soils (Lambrechts et al., 2019). The choice of this niche was deliberate. Bacterial richness in Antarctic soils is strongly conditioned by moisture and by the presence of plants and birds (Friedmann, 1993; Teixeira et al., 2013), and the rhizosphere of one of the only two vascular plants of the continent concentrates a comparatively nutrient-rich and competitive habitat in which antagonistic traits are more likely to be selected.

The screening design proved as informative as the isolates themselves. Of the 57 isolates assayed, 44 antagonized *B. cinerea* at 22 °C but only 28 retained that activity at 0 °C, and the ranking of the isolates was only partly conserved between the two regimes: values close to 60% at 22 °C corresponded to anything between 12 and 78% at 0 °C, and the five isolates selected here reached inhibition above 70% only at low temperature. A conventional screen performed at mesophilic temperature would therefore have ranked several of these strains as unremarkable. The reference antagonists make the same point from the opposite direction: *B. subtilis* and *B. amyloliquefaciens* inhibited the three *B. cinerea* strains by around 55% at 22 °C, comparable to the Antarctic isolates, yet showed no detectable inhibition at 0 ± 2 °C. This behavior reproduces the reported inability of commercial *Bacillus*-based formulations to operate at cold-chain temperatures and, within a single experiment, illustrates the gap that cold-active antagonists are intended to fill.

The five isolates with the strongest antagonism against *B. cinerea* belonged to the genus *Pseudomonas* and shared 98.58% 16S rRNA identity with the type strain *P. yamanorum* 8H1ᵀ, described in 2015 from isolates obtained on the subantarctic island of Ushuaia, Argentina (Arnau et al., 2015). Notably, 8H1ᵀ and other reported strains of this species have all been recovered from cold soils, including New Brunswick, Alaska, and Canadian grassland, so the association of our isolates with a cold-adapted lineage is coherent with their psychrotolerant phenotype. Because 16S rRNA identity is insufficient to delineate species within *Pseudomonas*, the 16S rRNA phylogeny placed AN3A02 within the *P. gessardii* subgroup (Figure 3A), and genome-scale analyses were used to test this placement, as recommended (Mulet et al., 2010; Gomila et al., 2015). GTDB-Tk phylogenomics placed strain AN3A02 within the recently proposed genus *Aquipseudomonas* (Rudra and Gupta, 2024), within a tight monophyletic clade with *Pseudomonas* sp. ATCC PTA-122608 and closest to the *P. yamanorum* cluster (Figure 4). Consistent with this placement, ANI values fell below the species boundary against every validly named reference genome, including the closest relative *P. yamanorum* LMG 27247 (94.89%), and in silico DDH against the same type strain (57.9% for AN3A02, 57.8% for ATCC PTA-122608) fell well below the 70% threshold for species co-specificity, while ANI between AN3A02 and ATCC PTA-122608 (98.92%) exceeded the species boundary, indicating they belong to the same, still undescribed species. Taken together, these results indicate that strain AN3A02 represents a putative novel species closely related to *P. yamanorum*. We refer to it here as *Pseudomonas* sp. AN3A02, following current usage for the strain, while noting that genome-scale phylogenomics places it within the recently proposed genus *Aquipseudomonas*, whose broader taxonomic adoption relative to *Pseudomonas sensu* stricto remains to be established.

The identical REP, ERIC, and BOX-PCR fingerprints obtained for the five isolates were unexpected, given that they were recovered from two different sampling sites and are consistent with a clonal population. Rep-PCR is widely used to discriminate *Pseudomonas* strains at the species level (Versalovic et al., 1991; Louws et al., 1994), and the absence of detectable polymorphism among our isolates justified focusing the whole-genome analysis on a single representative strain. The recovery of one genotype from two separate sites is consistent with the low diversity and the strong dispersal component described for Antarctic soils, where wind and migratory birds redistribute microbial propagules across ice-free areas (Friedmann, 1993; Teixeira et al., 2013) and where a successful genotype may dominate the cultivable fraction of a given niche. It should be noted, however, that rep-PCR resolves clonal complexes rather than individual genomes, so subtle differences among the five isolates cannot be ruled out until each is sequenced.

Phenotypic profiling was consistent with the molecular data: all isolates displayed the short-rod morphology, a single polar flagellum, fluorescence on King medium, and a 0–30 °C growth range typical of fluorescent pseudomonads, while the API-ZYM, hemolysis, and CAS results discriminated AN3A02 from its closest relatives by at least three differential characters. The comparison with the closest relative is informative in this respect. *P. yamanorum* 8H1ᵀ has a reported growth range of 4–35 °C, whereas the isolates described here grew from 0 to 30 °C, that is, they extend growth into the range at which fruit is stored while losing the upper end of the mesophilic range (Arnau et al., 2015). This displacement of the cardinal temperatures, together with the differences in esterase, lipase and β-glucosidase activities, separates the isolates from 8H1ᵀ by more than the characters conventionally required and agrees with the genomic indices that place AN3A02 outside the species. All five isolates also produced siderophores on CAS plates, a trait directly implicated in the antagonism of fluorescent pseudomonads through competition for iron and a plausible contributor to the inhibition observed in dual culture (Schwyn and Neilands, 1987). This phenotype has a genomic counterpart; the AN3A02 genome carries two distinct pyoverdine-like siderophore pathways (Regions 1.3 and 12.1, Table 3), consistent with a high-affinity, partly redundant iron-acquisition strategy that would favor competitive exclusion of the pathogen for essential trace metals under the nutrient-poor, cold conditions of postharvest storage.

From an applied perspective, the most relevant observation is that the five isolates retained strong inhibitory activity at 0–2 °C and were equally effective against the fenhexamid-resistant strain 7 ac and the multidrug-resistant strain PN2 as against the susceptible reference strain. Cold-active antagonism is precisely the property that conventional biocontrol agents lack and that is required to protect fruit during refrigerated storage and transport. The retention of activity against multidrug-resistant *B. cinerea* further suggests a mode of action distinct from the single-site targets to which these strains have evolved resistance, a desirable feature for integrated management strategies aimed at slowing the emergence of resistance (Hahn, 2014; Fisher et al., 2018).

The genomic analysis provides a plausible mechanistic basis for these observations. The AN3A02 genome encodes siderophore biosynthesis and, among its 402 annotated subsystems, genes for the acquisition of inorganic compounds, for the alleviation of abiotic stress in plants, and for the synthesis of phytohormones, antibiotic molecules, and volatile and aromatic compounds, a functional profile typical of rhizobacteria with a plant-beneficial lifestyle (Poblete-Morales et al., 2020). Siderophore production was confirmed phenotypically (CAS assay) under standard conditions, whether iron scavenging remains active at 0 °C was not tested and remains to be established. Accordingly, the cold-active antagonism observed here is attributed primarily to volatile compounds, which were directly assayed at 0 °C, rather than to siderophore-mediated iron competition. Nonribosomal peptide synthetases and polyketide synthases assemble many of the antifungal metabolites of the genus, and the 10 biosynthetic gene clusters predicted by antiSMASH in the AN3A02 genome (Table 3) show that this biosynthetic machinery is well represented rather than a marginal prediction. Region 16.1 displays similarity to the tolaasin I/F cluster. Tolaasins are cyclic lipopeptides (cLPs) with well-documented pore-forming and membranolytic activity against fungal pathogens, which would provide a direct molecular mechanism for the hyphal-growth inhibition observed in the dual-culture assays. Fluorescent pseudomonads are also well-known producers of volatile antimicrobial compounds and of hydrogen cyanide under GacA control (Blumer et al., 1999; Hunziker et al., 2015), and volatile metabolites are especially attractive for postharvest use because they act at a distance and do not require contact with the fruit surface. These remain, however, in silico predictions.

Independent chemical evidence for this volatile potential already exists for the very same strain. Using headspace SPME coupled to GC–MS, Ulloa et al. (2023) resolved a diverse volatile profile from *Pseudomonas* sp. AN3A02, comprising 45 compounds distributed across twelve chemical classes, several of which have previously been reported as antifungal, and showed that this volatile fraction exerts a fungistatic effect on *B. cinerea*, reducing gray-mold severity on blueberries by roughly 71% under postharvest conditions. Notably, several of these volatiles, including sulfur compounds such as dimethyl disulfide and dimethyl trisulfide, continued to be released throughout storage at 0 °C, indicating that emission is not abolished at cold-chain temperatures. Among these persistent volatiles, the ketone 2-nonanone, 1-octanol and methanethiol, together with the disulfides above, have antifungal activity documented in previous reports and are therefore the strongest candidate effectors of the cold-active phenotype reported here; these individual compounds were not, however, tested in pure form against *B. cinerea* in the present work or by Ulloa et al. (2023), so their specific causal contribution remains to be confirmed. HCN, by contrast, was not detected in the headspace of AN3A02, so the cold-active phenotype is more parsimoniously attributed to these recovered volatiles than to cyanide. This prior characterization corroborates, from an analytical standpoint, the volatile-producing capacity inferred here from the genome. Our contribution is complementary, it places this volatile repertoire within the cold-active phenotype and its efficacy against fungicide-resistant strains and the taxonomic novelty of the strain, and broadens the biosynthetic context beyond the compounds resolved by GC–MS. The present study was designed to characterize the strain rather than to isolate the individual volatile effector, the compound-level profile of AN3A02 has already been resolved by Ulloa et al. (2023), and the definitive identification of the specific volatile(s) responsible for inhibition at 0 °C, together with a quantitative comparison of their emission at 0 °C and 22 °C, remains an objective for future work.

A first functional test of these predictions is provided by the volatile assays. When *Pseudomonas* sp. AN3A02 and *B. cinerea* were confronted in sealed two-compartment plates, sharing only the headspace, the bacterium reduced mycelial growth and suppressed the formation of sclerotia and conidiophores (Figures 6A,B), because the compartments never come into contact, this activity can be ascribed specifically to volatile compounds, which is consistent with the genome-based prediction of a volatile-producing machinery and indicates that at least part of the antagonism is independent of iron competition or diffusible metabolites. The effect extended to fruit under simulated cold-chain storage, where the volatiles of AN3A02 lowered gray-mold incidence from 51.7 to 1.7% on blueberries and from 100 to 30.0% on table grapes, outperforming the SO₂ emitter used as the postharvest standard and confining the residual infections to the inoculation site (Figures 6C–H). Volatile-mediated control is particularly attractive for the cold chain because it acts at a distance and does not require contact with the fruit (Rahman F. U. et al., 2024). The present data show that this activity persists at 0 °C against a multidrug-resistant strain and narrows the outstanding question to the chemical identification of the volatiles involved. This persistence, together with the growth range of the isolates (0–30 °C; Table 2), indicates that inhibition at 0 °C reflects active bacterial metabolism at that temperature rather than the carry-over of metabolites preformed during pre-culture, since an effect sustained over several weeks is difficult to reconcile with a finite pool of pre-formed compounds and instead implies continued volatile production under cold conditions. A direct, time-resolved quantification of bacterial growth and metabolic activity during cold volatile emission was beyond the scope of this study and remains an objective for future work. Although the AN3A02 genome encodes hydrogen-cyanide operons (Table 3), HCN was not recovered from its headspace and is therefore an unlikely effector, directing attention instead to the cold-persistent volatiles identified above (Ulloa et al., 2023).

Several limitations frame these conclusions. Although the antagonism was confirmed on inoculated fruit under simulated cold-chain conditions, these assays were carried out at laboratory scale, so validation under commercial packing and storage conditions is still required before any practical projection. In addition, the two-compartment design establishes a volatile-mediated contribution to the antifungal phenotype but does not identify the individual compound responsible. Resolving the specific effector and its dose–response was beyond the scope of this characterization study and constitutes a defined direction for future work. Biosafety must likewise be established before any applied use: although the isolates derive from an Antarctic environmental niche and belong to the *P. fluorescens* group (*P. gessardii* subgroup) rather than to the *P. aeruginosa* complex, and although in our assays the bacterium acts at a distance without contacting the fruit, we detected β-hemolysis on blood agar and did not perform formal safety testing. A dedicated biosafety assessment, including characterization of this hemolytic activity, antibiotic-resistance profiling, and cytotoxicity/pathogenicity testing, is therefore a necessary prerequisite, and the strains are presented here as characterization-stage candidates rather than as ready-to-use biocontrol agents. Finally, the present results nonetheless identify a defined, genetically characterized set of Antarctic *Pseudomonas* isolates as candidate biocontrol agents against fungicide-resistant *B. cinerea* under postharvest conditions and provide the taxonomic and genomic framework required for their further development.

## 5 Conclusion

Rhizosphere soil of the Antarctic vascular plant *D. antarctica* yielded cultivable bacteria with pronounced antifungal activity against *Botrytis cinerea* at temperatures relevant to postharvest storage. Five rep-PCR-indistinguishable *Pseudomonas* isolates inhibited mycelial growth by ≥70% at 0–2 °C and were equally active against fungicide-resistant and multidrug-resistant strains. Polyphasic and genome-based characterization of the representative strain AN3A02 placed it within the *P. gessardii* subgroup as a putative novel species and revealed a secondary-metabolite biosynthetic potential consistent with its antagonism. Our assays further point to a contribution of volatile compounds that remain active at 0 °C: in a contact-free system the isolate was associated with a reduced formation of the resistance and reproductive structures of *B. cinerea in vitro*, and on inoculated blueberries and table grapes its volatiles were associated with a lower incidence of gray mold under simulated cold-chain storage, comparing favorably with the SO₂ emitter used as the postharvest standard. These cold-adapted Antarctic *Pseudomonas* strains may represent promising candidates for the biological control of gray mold along the fruit cold chain. Identifying the volatiles involved and establishing their relative contribution, together with validation on a larger scale and under commercial conditions, and the formal taxonomic description of the novel species, would be needed before these strains could be developed into a biocontrol product.

## Data Availability

The data presented in the study are deposited in the NCBI GenBank repository, accession number JAARNI000000000 (BioProject PRJNA613530, BioSample SAMN14408689, SRA SRR11362444).

## Footnotes

1
https://ggdc.dsmz.de
