# Supplementary material for: Genomic and phenotypic characterization of Antarctic Pseudomonas/Aquipseudomonas sp. strains with cold-active antifungal activity against Botrytis cinerea
Source: Front Microbiol. 2026 Sep 23;17:1969102. doi: 10.3389/fmicb.2026.1969102 (PMC13642843; doi:10.3389/fmicb.2026.1969102)
Supplement: Supplementary file 1 [file Supplementary_file_1.DOCX]

Supplementary Material

# 1 Supplementary Figures and Tables

## 1.1 Supplementary Figures

**Supplementary Figure S1.** Transmission electron microscopy (TEM) of the representative strain Pseudomonas sp. AN3S0, showing the short-rod morphology and single polar flagellum characteristic of the five Antarctic isolates.

## 1.2 Supplementary Table 1

| Table S1. Mean percentage inhibition (± SD) of *B. cinerea* B05.10 radial mycelial growth by Antarctic *Pseudomonas* isolates at 22°C and 0°C. corresponding to Figure 1 | | | | |
| --- | --- | --- | --- | --- |
|  |  |  |  |  |
| **Isolate** | **Mean 22°C (%)** | **± SD 22°C** | **Mean 0°C (%)** | **± SD 0°C** |
| AN1A01 | 40.5 | 8.6 | 18.7 | 7.2 |
| AN1A02 | 49.8 | 7.5 | 26.3 | 5.2 |
| AN1A03 | 52.2 | 4.7 | 29.2 | 6.8 |
| AN1A11 | 52.2 | 4.5 | 30.7 | 9.0 |
| AN1A13 | 60.6 | 8.6 | 37.3 | 3.8 |
| AN1A14 | 48.5 | 8.0 | 23.1 | 10.9 |
| AN1A15 | 0.0 | 0.0 | 22.9 | 4.9 |
| AN1A23 | 55.0 | 2.7 | 0.0 | 0.0 |
| AN1A25 | 0.0 | 0.0 | 44.2 | 5.8 |
| AN1A26 | 42.6 | 9.5 | 32.9 | 8.3 |
| AN1A32 | 0.0 | 0.0 | 11.6 | 7.1 |
| AN1A33 | 0.0 | 0.0 | 14.3 | 7.2 |
| AN1A888 | 54.4 | 5.5 | 0.0 | 0.0 |
| AN2A01 | 50.2 | 5.9 | 0.0 | 0.0 |
| AN2A04 | 50.8 | 8.1 | 0.0 | 0.0 |
| AN2A05 | 54.8 | 0.3 | 35.4 | 6.9 |
| AN2A06 | 45.1 | 8.3 | 0.0 | 0.0 |
| AN2A33 | 0.0 | 0.0 | 14.7 | 4.4 |
| AN2B01 | 56.7 | 2.2 | 32.6 | 1.2 |
| AN2B04 | 51.8 | 7.2 | 29.2 | 7.5 |
| AN2B06 | 61.4 | 5.1 | 76.1 | 1.6 |
| AN2B07 | 62.0 | 7.0 | 78.0 | 2.9 |
| AN2B08 | 0.0 | 0.0 | 11.8 | 1.0 |
| AN2B12 | 59.0 | 2.1 | 25.2 | 9.1 |
| AN2B20 | 47.3 | 3.0 | 27.5 | 8.9 |
| AN2B30 | 49.5 | 6.6 | 17.6 | 8.3 |
| AN3A01 | 63.3 | 4.7 | 32.1 | 8.2 |
| AN3A02 | 58.6 | 4.7 | 75.0 | 3.2 |
| AN3A03 | 51.0 | 5.3 | 28.2 | 1.3 |
| AN3A04 | 58.0 | 2.8 | 21.0 | 9.5 |
| AN3A05 | 58.3 | 2.6 | 70.8 | 3.8 |
| AN3A801 | 0.0 | 0.0 | 26.6 | 4.4 |
| AN3A803 | 40.6 | 13.4 | 0.0 | 0.0 |
| AN3A804 | 42.5 | 6.2 | 0.0 | 0.0 |
| AN3B04 | 44.4 | 4.6 | 21.6 | 4.8 |
| AN3B12 | 0.0 | 0.0 | 32.1 | 8.2 |
| AN3B21 | 46.3 | 8.2 | 17.4 | 1.1 |
| AN3B22 | 0.0 | 0.0 | 17.1 | 6.2 |
| AN3B23 | 58.3 | 8.0 | 76.6 | 1.0 |
| AN3B24 | 41.3 | 2.7 | 49.8 | 3.8 |
| AN3B25 | 42.9 | 8.1 | 18.9 | 9.7 |
| AN3B30 | 0.0 | 0.0 | 30.4 | 8.6 |
| AN3B34 | 58.7 | 4.5 | 23.9 | 1.6 |
| AN3B39 | 47.3 | 6.7 | 26.1 | 1.6 |
| *B. a* | 57.2 | 7.6 | 0.0 | 0.0 |
| *B. s* | 54.4 | 7.9 | 0.0 | 0.0 |
| Values represent mean ± standard deviation of the percentage of inhibition of radial mycelial growth. as plotted in Figure 1. *B. a. Bacillus amyloliquefaciens*; *B. s. Bacillus subtilis* (reference biocontrol strains) | | | | |
